# Psoralen as a Photosensitizers for Photodynamic Therapy by Means of In Vitro Cherenkov Light

**DOI:** 10.3390/ijms232315233

**Published:** 2022-12-03

**Authors:** Lisa Hübinger, Roswitha Runge, Tobias Rosenberg, Robert Freudenberg, Jörg Kotzerke, Claudia Brogsitter

**Affiliations:** Department of Nuclear Medicine, University Hospital Carl Gustav Carus, Technische Universität Dresden, 01307 Dresden, Germany

**Keywords:** psoralen, Re-188, plasmid DNA, FaDu cells, Cherenkov light

## Abstract

Possible enhancements of DNA damage with light of different wavelengths and ionizing radiation (Rhenium-188—a high energy beta emitter (Re-188)) on plasmid DNA and FaDu cells via psoralen were investigated. The biophysical experimental setup could also be used to investigate additional DNA damage due to photodynamic effects, resulting from Cherenkov light. Conformational changes of plasmid DNA due to DNA damage were detected and quantified by gel electrophoresis and fluorescent staining. The clonogene survival of the FaDu cells was analyzed with colony formation assays. Dimethyl sulfoxide was chosen as a chemical modulator, and Re-188 was used to evaluate the radiotoxicity and light (UVC: *λ* = 254 nm and UVA: *λ* = 366 nm) to determine the phototoxicity. Psoralen did not show chemotoxic effects on the plasmid DNA or FaDu cells. After additional treatment with light (only 366 nm—not seen with 254 nm), a concentration-dependent increase in single strand breaks (SSBs) was visible, resulting in a decrease in the survival fraction due to the photochemical activation of psoralen. Whilst UVC light was phototoxic, UVA light did not conclude in DNA strand breaks. Re-188 showed typical radiotoxic effects with SSBs, double strand breaks, and an overall reduced cell survival for both the plasmid DNA and FaDu cells. While psoralen and UVA light showed an increased toxicity on plasmid DNA and human cancer cells, Re-188, in combination with psoralen, did not provoke additional DNA damage via Cherenkov light.

## 1. Introduction

UV light is established for therapeutic purposes for numerous skin diseases. UVB light is especially used for treating psoriasis and UVA light can be performed when treating atopic eczema [1,2,3]. Since light can activate primarily non-toxic substances to phototoxic ones, it is a so-called photosensitizer. The typical procedure during photodynamic therapy (PDT) activates a photosensitizer via the radiation of visible light. The therapeutic effect is either performed directly by effects on the DNA or indirectly by the formation of singlet oxygen or reactive oxygen species such as hydrogen peroxide or superoxide anions. The small penetration depth of light in the visible and the UV scope, however, limits the applicability of PDT to surface lesions and does not allow for the treatment of solid tumors inside the body. These limitations could be overcome by Cherenkov light (CL), which can be produced by diagnostically or therapeutically used radioisotopes (e.g., Ga-68, Re-188, or Y-90) [4,5,6]. CL is already used diagnostically, for example, in visual imaging [3,7,8,9]. This features a new therapeutic approach: depositing phototoxic substances inside the tumor and activating these via CL in addition to internal or external irradiation. Thereby, a local combination of radiotoxicity and phototoxicity induces a more profound treatment [10,11]. This vision of PDT is promising; however, the mechanism is not yet illuminated completely. Recent research follows up with different methods and studies that confirm this vision. Yoon et al. [12] used trioxsalen (a psoralen derivative) and irradiated melanoma and breast cancer cells. The CL was created by external radiation, producing CL in a solid water block.

This work investigated this idea in a nuclear medicine setting. In doing so, the technique postulated by Yoon et al. was combined with the precision of nuclear medicine directly targeting tumor volume, even deep inside the patient. Our proof-of-principle study expands on the research by Yoon et al. by implementing CL via the radioisotope Re-188 in comparison to external radiation and investigated the photoactivation of psoralen by the Re-188-produced CL. Not only this new method, but also an additional setting with protein-free plasmid DNA, were tested for performance and suitability. The results were compared with the phototoxicity of a defined wavelength scope.

This simple setup lays the groundwork for follow-up studies approaching PDT enhancement with CL, radioisotopes, and psoralen, along with demonstrating fundamental methods in photoactivation and the measuring of CL in a nuclear medicine approach.

## 2. Material and Methods

### 2.1. Plasmid DNA and Cell Culture

#### 2.1.1. Plasmid DNA

A pUC19 plasmid with 2686 base pairs with a molar mass of 1.75 × 10^6^ Dalton was used (New England Biolabs, Ipswich, UK). The DNA stock solution was adjusted to a concentration of 0.1 µg/µL via a TE buffer (10 mM Tris-HCl, 1 mM EDTA, pH 7.5). All plasmid DNA solutions contained ≥95% supercoiled plasmid DNA. To obtain linear marker plasmid DNA, pUC19 was enzymatically treated with BamHI (Invitrogen, Karlsruhe, Germany).

#### 2.1.2. FaDu Cells

FaDu cells are squamous cell carcinoma cells of the pharynx. They were retained via biopsy in 1968 and have since grown as a monolayer [13]. The sub cell line FaDu_DD_ has been used in radiobiological experiments since the 1980s and possesses a doubling time of about 18 h in the exponential phase [14]. FaDu cells (ATCC^®^ HTB-43^TM^) originate from an undifferentiated human squamous cell carcinoma. In our experiments, the sub cell line FaDu_DD_, kindly provided by the Department of Radiotherapy and Radiation Oncology, Medical Faculty, Technical University Dresden, was used. The cells were maintained in Dulbecco’s minimum Essential medium (DMEM) containing 2% Hepes buffer, 1% of non-essential amino acids, 1% sodium pyruvate, and 10% fetal calf serum.

### 2.2. Psoralen and DMSO

Psoralen (CAS: 66-97-7, purity ≥99%, Sigma Aldrich, Darmstadt, Germany) is the basic compound of the linear furanocoumarins and shows photosensitive properties. At first, a non-covalent bond is formed with the DNA. Due to UV irradiation, a covalent bond between psoralen and a pyrimidine base (preferentially thymine) is formed via cycloaddition [15]. With further UV irradiation, more interactions between the psoralen monoadduct and the pyrimidine base can occur, so that crosslinks between DNA strands are formed. Furthermore, psoralens react with other cellular structures such as proteins or lipids. The general absorption maximum of psoralen lies between 320 nm and 400 nm, while the psoralen used in this publication has its maximum at 355 nm [16]. Dimethyl sulfoxide (DMSO, CAS: 67-85-5, purity = 99.9%, Sigma Aldrich, Darmstadt, Germany) was used as a radical scavenger in a final concentration of 0.2 M.

### 2.3. Light and Radioactivity

The UV irradiation took place with a UV lamp (Type 022.9230, LAMAG, Berlin, Germany) in the UVC region (*λ* = 254 nm) and in the UVA region (*λ* = 366 nm) of the spectrum. The irradiation times varied between 1 and 12 min (254 nm) and 10 to 120 min (366 nm). For a 5 cm distance between the probes and the light source, the spectro-radiometric measurements showed an irradiance of 20.5 W/m^2^ and 14.0 W/m^2^ for *λ* = 366 nm and *λ* = 254 nm, respectively [5].

Re-188 was extracted from a tungsten/rhenium generator (Isotope Technologies Garching GmbH, Garching, Germany). The high-energy beta particles of Re-188 (maximum beta energy *E_β,max_* = 2.12 MeV, mean energy 765 keV, and maximum penetration depth of 1.05 cm in tissue) can be used therapeutically, whereas its gamma radiation (energy 155 keV, intensity 15.8%) is used for imaging. 

During its decay, Cherenkov light with a yield of 35 photons per decay event is produced [4]. Its physical half-life is 16.98 h and Re-188 has an average linear energy transfer (LET) of 0.19 keV/µm.

The irradiation geometry used in Monte Carlo simulations for plasmid DNA in a 20 µL volume resulted in 7.7 Gy/(1 MBq * 1 h) and 120 Gy/(1 MBq * 24 h), respectively. Thus, cells in a 6-well plate with a 2 mL volume receive 2 Gy/(1.37 MBq * 24 h).

### 2.4. Agarose Gel Electrophoresis, Colony Formation Assay and Cherenkov Light

#### 2.4.1. Agarose Gel Electrophoresis

Plasmid probes with each 200 ng DNA (0.1 µg/µL) were incubated with varying volumes of chemical noxa or radionuclide solutions in 1.5 mL micro tubes (Eppendorf, Hamburg; Germany) in a total volume of 20 µL. After treating the plasmid probes, 10 µL of each DNA solution was mixed with 1.25 µL loading buffer (Invitrogen, Karlsruhe, Germany).

The probes were pipetted into the pocket of a 1.4% agarose gel in Tris-Acetat-EDTA buffer (TAE, Sigma, Darmstadt, Germany). The electrophoresis (Bio-Rad Laboratories GmbH, Munich, Germany) took place on ice with a voltage of 4 V/cm over 120 min. Due to differences in the mobility of the plasmid conformations, supercoiled (SC), open circular (OC), and linear (L) conformations can be distinguished. 

The agarose gel was stained with an ethidium bromide solution (0.5 µg/mL) and the plasmid DNA was detected with a UV transilluminator (DIANA III Digital Imaging System, Straubenhardt, Germany). The fluorescence intensities of the DNA bands were quantified with the analysis software Fiji [17] and interpreted as undamaged native plasmid DNA (SC conformation), DNA single strand breaks (OC form) as well as DNA double strand breaks (L conformation).

#### 2.4.2. CL Verification

CL was verified in the same system that the electrophoresis used (Bio-Rad Laboratories GmbH, Munich, Germany). The included charge-coupled device camera detects the CL produced by Re-188, since it can detect light in the wavelength region of the Cherenkov spectrum. Each verification image shows the intensity of light recorded by the camera over 10 min. The ImageLab software provides the opportunity to analyze different areas and evaluates the integrated volume light intensity. However, this analysis is not quantitative and only works as a general verification and holds for comparisons between different setups.

#### 2.4.3. Colony Formation Assay

To determine the clonogene survival, a colony formation assay was used [18]. After 24 h, the cells were stripped, an aliquot for each dose point (or UV irradiation time) of the cell suspension was taken for the colony formation test in T25 culture flasks, and finally placed in an incubator for 9 days. To stop colony formation, the cells were fixed with ethanol 80% v/v and stained with crystal violet. The counting of the colonies was performed on a microscope (magnification 25×). The plating efficiency and the survival rate (or survival fraction) was calculated for both the irradiated and unirradiated cells [18].

### 2.5. Statistics

All results are shown as the average as well as the SEM (standard error of the mean) of the plasmid DNA and the pooled standard deviation (SD_pooled_) of the cell experiments based on three independent tests (each experimental setup was determined as a triplicate). To prove the statistical significance, a Student’s t-test was used. A difference between two independent samples was seen as significant if the probability of error was *p* ≤ 0.05. The statistical analysis was conducted with MS Office Excel.

## 3. Results

### 3.1. Plasmid DNA and Psoralen with UVA

Preliminary tests excluded that the incubation time (1 h or 24 h) of the plasmid DNA with psoralen without exposure to light influences the DNA integrity. Thus, small time differences in the sample processing can be neglected.

**Figure 1:**
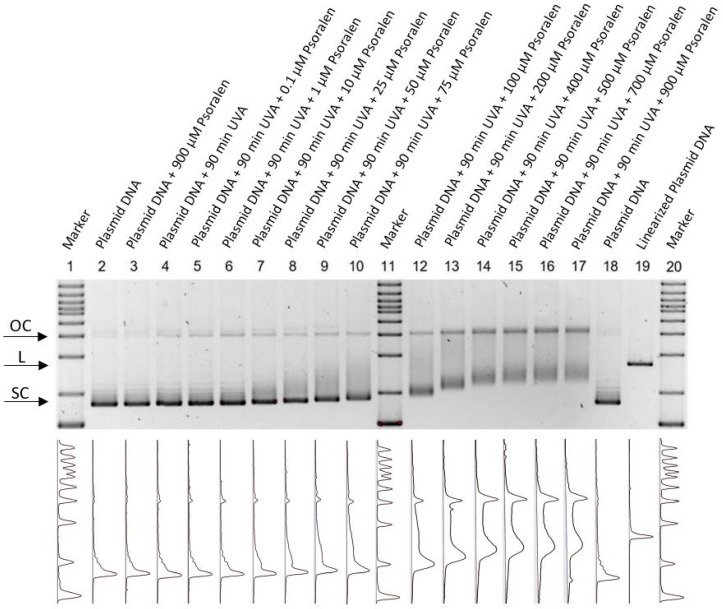
Fluorescent measurement of an agarose gel for combining psoralen with UV-366 nm. The aperture detects the intensity spectra (**bottom**) of the fluorescent gels and displays them visually (**top**). The figure is inverted, thus showing a dark color for high intensities. Since this measurement is not quantitative, the intensity scale only shows arbitrary units. Markers are placed in lanes 1, 11, and 20. Lane 2 and 18 show untreated plasmid DNA, whilst lane 19 contains enzymatically linearized plasmid as the control. In lanes 4–10 and 12–17, different psoralen concentrations (0, 0.1, 1, 10, 25, 50, 75, 100, 200, 400, 500, 700, 900 µM) were irradiated with UV-366 nm for 90 min. Each lane was normalized and shows the running behavior of the different setups. A higher concentration of psoralen showed a higher amount of open circular conformations (OCs). Lane 3 demonstrates non-UV-activated psoralen (900 µM).

The effect of increasing concentrations of psoralen with or without UVA exposure can be seen in Figure 1. The running behavior depends on the conformational change in each lane, thus supercoiled (SC), linear (L) and open circular (OC) conformations can be distinguished. Even though non-UVA-activated psoralen in the highest concentration did not show chemotoxicity, there was a change in the running behavior after light exposure, which can be explained by the uncoiling of the SC DNA and the formation of psoralen DNA monoadducts [19].

**Figure 2:**
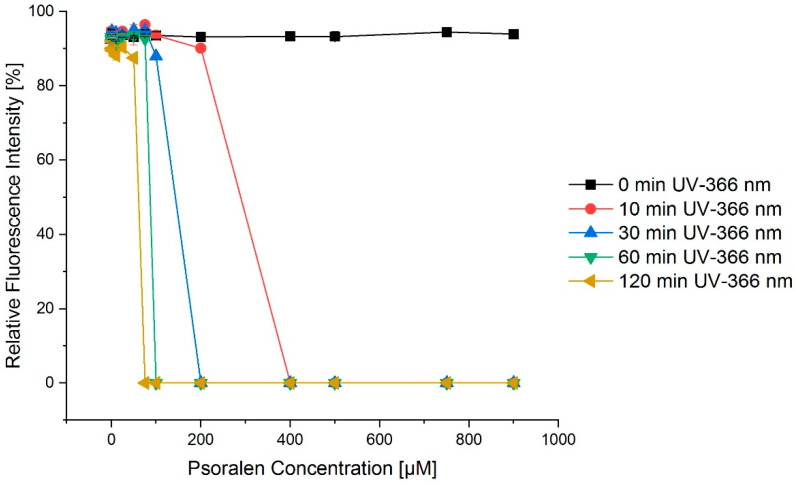
Exemplary concentration–effect relations for the combination of psoralen and varying irradiation times of UV-366 nm for the SC conformation. The longer the irradiation with UV-366 nm, the more intense the fractions of the open circular confirmation as a consequence of the SC conformation quickly dropping to zero. The error bars show the SEM.

A similar effect was visible for a constant concentration of psoralen and an increasing integral dose of UVA light achieved by longer irradiation times (Figure 2). When looking at the case of 30 min UV treatment and psoralen concentrations in the range of 100 µM to 500 µM, Figure 2 nicely shows that the fraction of SC conformations quickly dropped to zero, and thus the OC conformations increased.

**Figure 3:**
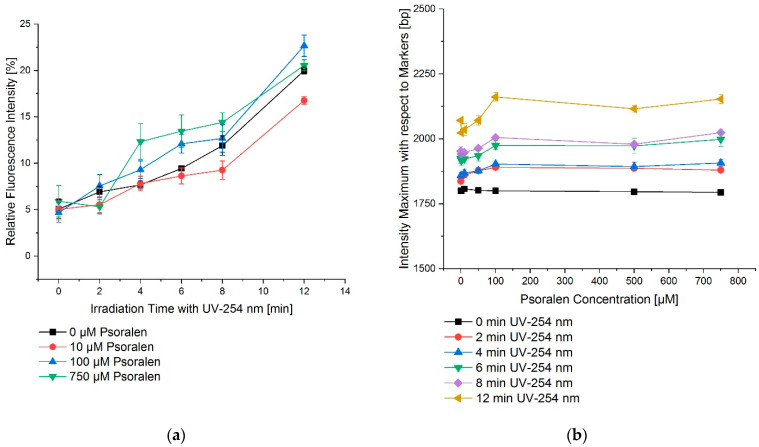
UV irradiation time–effect relations for the combination of psoralen with UV-254 nm. (**a**) OC fraction as a function of irradiation time, (**b**) OC fraction as a function of psoralen concentration. The effects are displayed as changes in the migration distances of DNA in relation to the markers bp. To increase the irradiation time, the effect as in relative fluorescence intensity also increased. However, this effect was nearly concentration independent. The error bars show the SEM.

Figure 3a depicts the dependency on the time of irradiation for UVC (254 nm). However, this effect could not be increased for varying concentrations of psoralen (Figure 3b).

**Figure 4:**
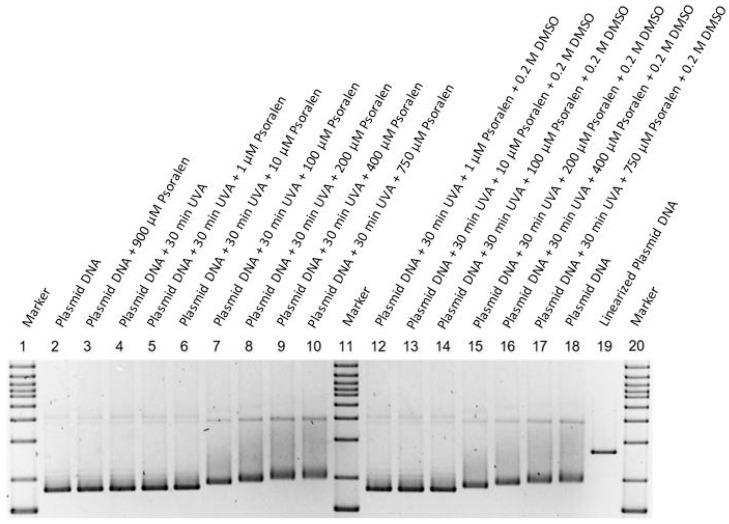
Fluorescent measurement of an agarose gel for combining psoralen with UV-366 nm with an additional 0.2 M DMSO for several lanes. The figure is inverted, thus showing a dark color for high intensities. Markers are placed in lanes 1, 11, and 20. Lane 2 and 18 show the untreated plasmid, whilst lane 19 contains the linear plasmid. In lanes 4–10 and 12–17, different psoralen concentrations (0, 1, 10, 100, 200, 400, 750 µM) were irradiated with UV-366 nm for 30 min, where an additional 0.2 M DMSO was present in lanes 12–18. Each lane was normalized and shows the running behavior of the different setups. In comparison to Figure 1, the additional DMSO did not produce a different behavior. Lane 3 demonstrates non-UV-activated psoralen (900 µM).

Psoralen was dissolved in DMSO (1% *v/v* ≡ 0.14 M DMSO) to obtain a stock solution of 1000 µM psoralen. To further investigate the additional effects of DMSO on the damage of DNA, the light exposure was repeated with and without DMSO (0.2 M) concentrations with varying concentrations of psoralen. However, in either way, there was no visible effect (Figure 4).

### 3.2. Plasmid DNA and Psoralen with Re-188

**Figure 5:**
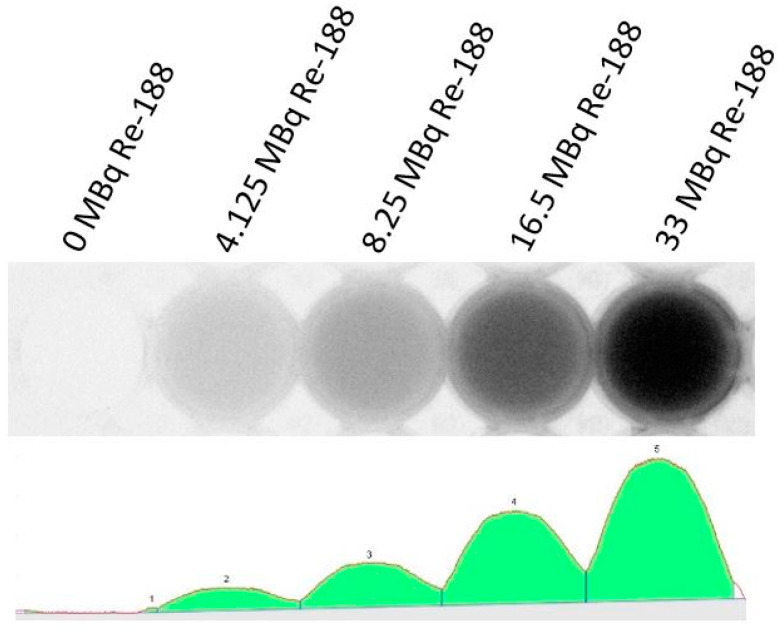
Light intensity of different amounts of Re-188 in a 24-well plate. The picture is inverted, thus, showing increasing intensities of light from left to right (f.l.t.r.: 0, 4.125, 8.25, 16.5, and 33 MBq). The spectrum (**bottom**) was detected by the camera and is shown visually at the top of the figure. If more Re-188 radioactivity is present, the larger the light intensity will be. All intensities were not quantifiable.

The CL yield of Re-188 is dose-dependent. Figure 5 shows this effect.

**Figure 6:**
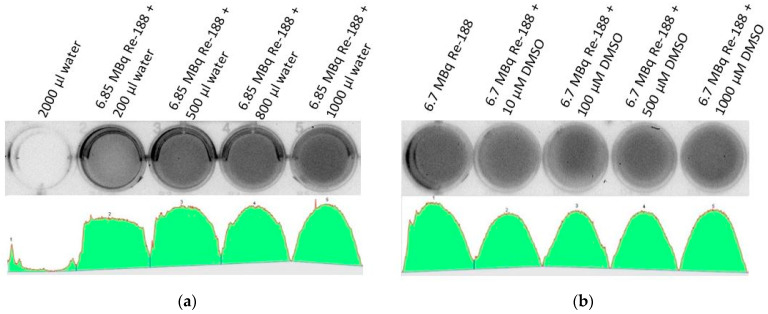
Light intensity of different setups of Re-188 and DMSO as preliminary investigations. The spectra show the detected light intensity in arbitrary units, thus, they are not quantifiable. (**a**) The first well did not contain radioactivity (2000 µL distilled water) and did not consequently show light intensity. The other wells contained a nearly constant amount of Re-188 (approximately 6.85 MBq) and varying volumes of water (f.l.t.r. 200, 500, 800, and 1000 µL). More light intensity was seen for larger water volumes. The spectrum showed that there was no linear behavior between the volume of the medium and the light intensity. However, a saturation was reached for a volume large enough to produce a 1 cm water column (depends on size of well). Measured light intensity: 7 Mio (background, no activity), 22 Mio, 27 Mio, 28 Mio, 29 Mio integral light volume intensity in arbitrary units for each well. (**b**) All wells contained a constant amount of Re-188 (approximately 6.7 MBq), varying amounts of DMSO, and additional water to reach a constant volume of 2000 µL. From left to right, an increasing amount of DMSO was added to the wells (0, 10, 100, 500, and 1000 µM). The same light intensity was seen for every DMSO concentration, since it is not photo-activated by Re-188. Measured light intensity f.l.t.r.: 28 Mio, 26 Mio, 26 Mio, 27 Mio, 27 Mio integral light volume intensity in arbitrary units for each well.

Additionally, Figure 6 shows the effect of varying volumes of water in combination with Re-188 visible in the measured light intensity. This is important since a certain volume of medium is needed so that the Re-188 can produce CL. For larger water volumes, an increase in light intensity was observed. A maximum volume (in the form of a 1 cm water column depending on the used setup) showed a saturating effect regarding the light intensity. This is due to the depth of the electrons in water producing the measured CL intensity. It has also been proven that DMSO does not have an influence on the light intensity, since it is not photo-activated by the resulting CL (Figure 6b).

**Figure 7:**
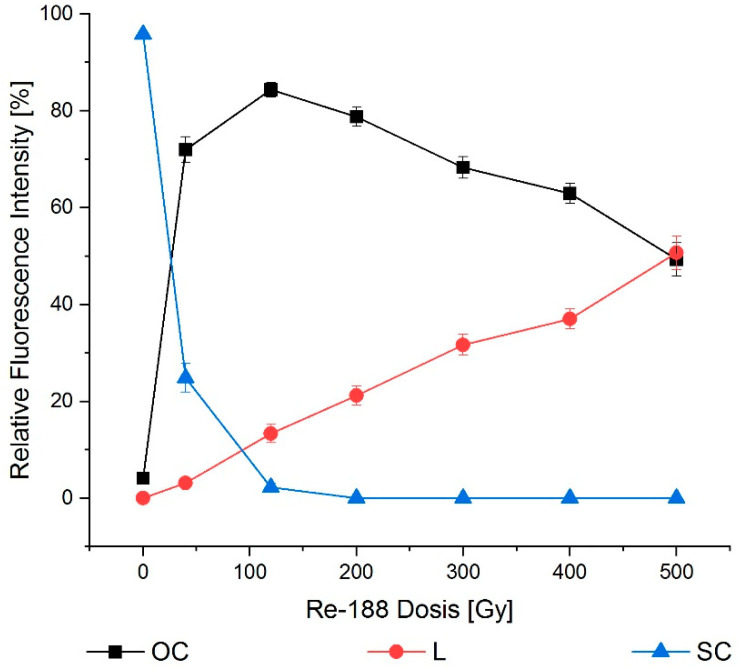
Dose–effect relation for Re-188 after 24 h incubation. The mere radiotoxicity of Re-188 had an influence on all conformations. Supercoiled (SC) transformed into open circular (OC) and eventually into linear (L) conformations for higher doses. The error bars show the SEM.

Mere Re-188-induced radiotoxicity showed a decrease in SC conformations, and thus an increase in the OC fraction. The highest dose showed a transformation of OC to the L plasmid (Figure 7).

**Figure 8:**
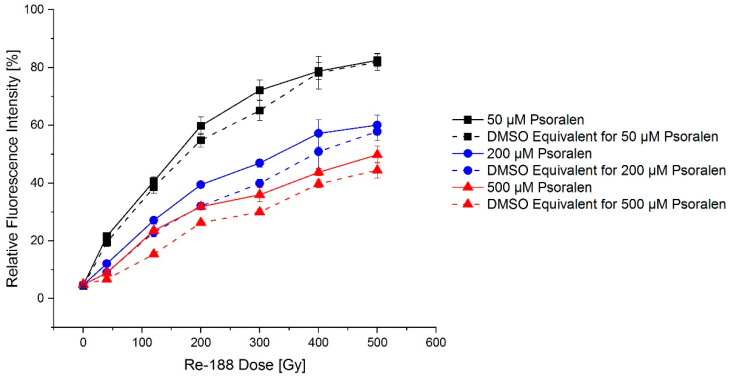
Exemplary dose–effect relation for Re-188 in combination with psoralen after 24 h incubation demonstrated an increasing fraction of open circular plasmid DNA. The influence of DMSO was tested. Psoralen produced a small additional effect, as visible in the difference between the continuous and dashed line. The error bars show the SEM.

Regarding the effects of psoralen, it has to be considered that psoralen is dissolved in DMSO, which is a radical scavenger and thus shows a protective effect on the radical-induced DNA damage. In Figure 8, equivalent amounts of DMSO with and without psoralen are compared. 

Small differences between the effects could be demonstrated with a slightly larger effect for psoralen compared to DMSO. Dose–wise comparisons were partially significant (e.g., at 200 Gy, 200 µM: *p* = 0.01).

### 3.3. Plasmid DNA and Psoralen with a Combination of UVA and Re-188

**Figure 9:**
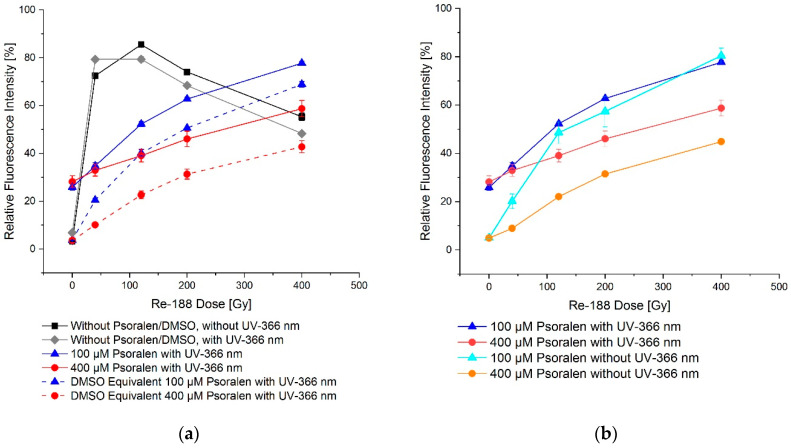
Dose–effect relation on plasmid DNA for Re-188 in combination with psoralen and with (**a**)/without (**b**) a 60-min UV-366 nm irradiation. (**a**) shows the additional fluorescence intensity of open circular conformation for mere radiotoxicity with/without UV-366 nm irradiation. There was a difference between the psoralen and the DMSO equivalent effect. In (**b**) the difference of the fluorescence intensity is shown by comparing UV-366 nm irradiation and no irradiation. The effect was larger with UV-366 nm irradiation. The error bars show the SEM.

For a combination of UVA and Re-188, a slightly more visible formation of single and double strand breaks was observed when psoralen and DMSO, respectively, were not present. When psoralen was present, the effects were weakened, but still stronger than in the case where only DMSO was added (Figure 9). 

### 3.4. FaDu Cells with Psoralen and UVA

In the course of the preliminary experiments, the impact of the time prior to incubation, meaning the time between the addition of psoralen and the UV irradiation on the clonogene survival was excluded. 

**Figure 10:**
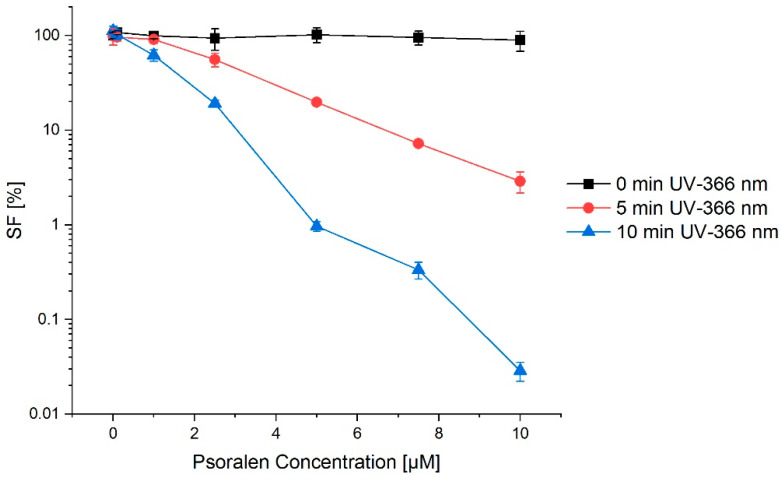
Concentration–effect relation for a combination of psoralen and UV-366 nm (after 24 h preincubation of psoralen in FaDu cells). The longer the irradiation with UV-366 nm, the lower the cell survival fraction (SF). This effect was enhanced by adding psoralen. Without irradiation, there was no difference in survival for any psoralen concentration. The error bars show the SD_pooled_.

Psoralen by itself is not toxic in the used concentration and does not affect the cell survival. The same holds for the light exposure. For a combination of UVA irradiation and the photosensitizer, a clear increase in cell damage was visible, depending on the psoralen concentration and the irradiation time (Figure 10).

### 3.5. FaDu Cells with Psoralen and Re-188

**Figure 11:**
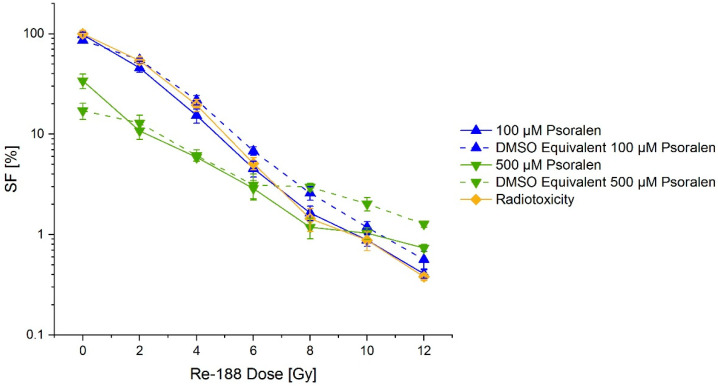
Concentration–effect relation for a combination of psoralen and Re-188 on the survival (SF) of FaDu cells (mere radiotoxicity is additionally shown). No significant difference between mere radiotoxicity and adding of psoralen was visible. The error bars show the SD_pooled_.

Doses of 0 Gy to 12 Gy were applied, and consequently the cell survival was reduced to 0.5%. A clear increase in the effect was not observed for higher concentrations of psoralen, even though there were small differences between some psoralen and DMSO concentrations and applied doses. This can be seen in Figure 11. 

## 4. Discussion

For some time, it has been issued that CL is not only usable for visual imaging [3,8,9] but can also be used for the photoactivation of photosensitizers [20]. Nonetheless, the experimental data have controversial opinions [5,21,22,23,24,25] and the essential principles are not clear. 

Psoralen is clinically well-established for photodynamic therapy (PDT) [2] and can be used on isolated plasmid DNA as well as intact cells. Plasmid DNA has several crucial advantages. Here, it works as a basic setup, which is needed to investigate the principle methods and procedures. There is no barrier in plasmid DNA, as there would be in cells. In addition, the control over scavengers is possible, which allows for an influence on the whole system. Additionally, plasmid DNA does not have repair mechanisms, thus the caused damage is not interfered with. Using these benefits, a more precise and controllable environment was created for the experiments. We expected to find the same effects in the cell model as described for the plasmid DNA. These can become clearer due to further cell structures (cell organelles, proteins) and the impairment of DNA repair due to the formation of crosslinks. 

The photochemical reaction of psoralen with the pyrimidine bases and the formation of psoralen monoadducts and crosslinks showed the well-examined and essential effects of the combination of psoralen with UVA (PUVA therapy) [19,26,27,28,29,30]. This again confirms the applicability of this concept.

UVA can be induced by photon irradiation in the megavolt range [10,12,31,32,33]. In a simple in vitro model, Yoon et al. showed that tumor cells incubated with psoralen showed a 10–20% lower survival fraction (SF) when exposed to light coming from megavolt irradiation, in addition to ionizing irradiation. This approach was adopted for the radionuclides. In plasmid DNA, it could be shown that psoralen is not toxic and does not damage the DNA. Only when exposed to light in the scope of the absorption maximum of psoralen (366 nm) did the interconnectivity of the DNA lead to a change in electrophoretic properties. However, only a small number of single strand breaks (SSBs) and no double strand breaks (DSBs) were observed. UVC (254 nm) showed a direct damage to plasmid DNA that could not be enhanced by prior incubation with psoralen nor prevented by scavenger DMSO (data not shown). 

The typical effect of ionizing radiation on DNA in the form of SSBs and DSBs on plasmid DNA is known [34] and was confirmed using Re-188 (Figure 7). The emission of high energetic electrons also generates CL. Thus, radioisotopes provide the opportunity to produce CL and are usable for PDT. Since psoralen is dissolved in 1% DMSO solution, which serves as a scavenger, the combined experiments showed a much lower damage of DNA than the control without psoralen. Yoon et al. did not go into detail on this aspect, since they did not have a control without psoralen, but only as an additional effect visible for exposed cells compared to the light shielded cells. In principle, a negative control was also possible for exposed radioisotopes when using isotopes with none or only very low emissions of CL (e.g., Lu-177 with 0.141 yield per decay) [4]. 

Even though there was no toxic effect by only psoralen or light (*λ* = 366 nm), the combination of both showed an energy- and concentration-dependent decrease in cell survival. Since these effects were observed in the presence of the DMSO (as the dissolvent for psoralen), scavenger-induced processes cannot be the source of this effect. 

The treatment of FaDu cells with Re-188 showed a dose-dependent reduction in the cell survival. There was a small additional cytotoxic effect visible for psoralen concentrations larger than10 µM compared to the DMSO equivalent combined with Re-188. This effect could be proven frequently. There was no increased cytotoxicity with increasing psoralen concentration and constant dose observable. Deviations for the highest psoralen concentration can be ascribable to the fact that only half of the solution was still in the culture medium, whilst the other half was the psoralen solution. The small fraction of culture medium implies a deficit of nutrition for the cells. Runge et al. [34] investigated the scavenging effect of DMSO on the PCCl3 cell line. The effect was the highest for a standard dose of 7.5 Gy and 0.2 M DMSO (*SF_+DMSO_/SF_-DMSO_* factor = 2.83). For DMSO concentrations of 0.01 mM and 0.05 mM, the SFs were 1.14 and 1.69, respectively. For a dose of 8 Gy and either 100 µM or 500 µM (corresponding to 0.013 mM and 0.065 mM DMSO, respectively) psoralen solution, the SFs resulted in values in the same magnitude: 1.79 and 2.06, respectively. Most likely, the deviation was due to the differences between the FaDu and the PCCl3 cell lines. 

In conclusion, an activation of psoralen by Re-188 is rather unlikely—the crosslinks are more complex and much harder to repair compared to the scavenger-induced DNA damage, so they have an extended effect on the cell survival. The SF stayed constant for psoralen concentrations >10 µM for an incubation with Re-188, while there was a clearly visible concentration-dependent decrease for the UV exposed psoralen incubated cells. Since there already are a variety of scavengers and enzymes for reactive oxygen species in the culture medium and the cells themselves, only a small influence of the DMSO solution on the biological end point is expected. This is confirmed by the fact that the SF only merely changed for the increasing DMSO concentrations, while a large amount of SSBs and DSBs could be depleted in the plasmid model.

For plasmid DNA, the CL yield was calculated to be 8.14 * 10^12^ photons for an irradiation with 4.22 MBq Re-188 over 24 h in 20 µL. In comparison, a 10-min UVA irradiation (*E* = 20.5 W/m^2^, *λ* = 366 nm, 5 cm distance, 0.09 cm^2^ cross-sectional area) induced 0.11 J, which corresponds to 1.04 × 10^17^ photons [5]. Due to the 10^4^-times lower light yield for an irradiation with Re-188, a measurable effect was not expected. The cell irradiation was similarly disadvantageous: a 5-min irradiation (*E* = 20.5 W/m^2^, *λ* = 366 nm, 5 cm distance, 9.6 cm^2^ cross-sectional area) had a 1.09 × 10^19^ photon yield, while 24 h of 8.22 MBq of Re-188 in 2 mL resulted in a 1.59 × 10^13^ photon yield. However, luminescence possibly takes up a large fraction of the light yield, which was not taken into account in this calculation [35]. Yoon et al. did not make an assumption on the photon yield in their experiments. The information on the CL irradiation with 4–8 µJ/m² per Gy in the spectrum of 250–850 nm [12] concluded in a photon yield in the magnitude of 10^14^ for a 6 MeV irradiation, resulting in a dose of 10 Gy. Additionally, Yoon et al. used a 3 cm block of solid water to create CL, whereas in the case of the irradiation in a 20 µL Re-188 solution, there was not enough volume to achieve the maximum Cherenkov yield of 34.9 photons per decay [4].

Hartl et al. [21] used Yttrium-90 (Y-90, a beta emitter) for PDT in combination with clinically established aminolevulinic acid (Prodrug by Protoporphyrin IX) and a modified tetraphenylporphyrin and observed small (first sample) and significant (second sample) differences. Even though Y-90 had a photon yield of 47 photons per decay (compared to 35 photons/decay for Re-188), it did not correspond to the high magnitudes. Another recent examination by Krebs et al. used the loss of the protective group on the kinase inhibitor AZD5438 as a model to prove the photoactivation [6]. A clear effect was observed for 419 MBq Y-90. A 14-h irradiation with a photon yield of 47.3 per decay corresponded to 6.6 × 10^15^ Cherenkov photons. Comparable 2-min irradiations with UVA corresponded to a photon number of 8.3 × 10^18^. 

Similar conclusions can be drawn for Kotagiri et al. [23], Quintos-Meneses et al. [36] and Chen et al. [37], who used F-18-FDG with 1.32 photons per decay, so that the photon yield was only at about 4% for Re-188 [5]. Due to their simulation calculations, Glaser et al. also concluded “that it is unlikely that the emission of Cherenkov light by radionuclides is usable source for phototherapy” [38]. A similar setting with higher activity of Re-188 could be investigated. On the other hand, a contrary conclusion was drawn by Pratt et al., where studies showed that CL was detectable in the human body and imaging was different for varying light and radioactivity. Since light is generated even in the depth of tissue this approach is suitable for PDT [39]. Additionally, a new approach compared CL imaging in the visible light range with short-waved infrared CL imaging, showing several advantages regarding the penetration depth and scattering effects [40]. Therefore, different explanations need to be found for the effects described in the literature. Only high activity concentrations of several hundred MBq/mL like those attained in selective internal radiation therapy showed a promising generation of Cherenkov photons by the exposed radionuclides.

## 5. Conclusions and Outlook

The phototoxicity of psoralen with light of different wavelengths (UVC: *λ* = 254 nm and UVA: *λ* = 366 nm) as well as the effect of Re-188, whose emitted beta radiation generates Cherenkov light with a yield of 35 photons per decay, was examined on plasmid DNA and a tumor cell line. Psoralen itself is not chemotoxic. Nonetheless, in combination with UVA (however, not with UVC), a concentration- and dose-dependent effect could be observed on plasmid DNA and intact cells. Thus, the models were suitable to prove phototoxicity. Both models showed a significant radiotoxicity for Re-188. However, there was no additional significant Cherenkov-induced phototoxicity observed when combined with psoralen. 

Psoralen derivatives with a higher efficiency [41] or other photosensitizers better fitting the emission spectrum of the Cherenkov light [42] could be used. Furthermore, nanoparticles that supply a light amplification or a wavelength shift toward the red spectrum could be examined [25,33,43,44]. Thereby, enhancement of the effect by a factor of 3000 has been described [45]. Magnetic nanoparticles could be shifted to the tumor region by external magnetic fields to increase the efficiency [46]. In addition, it would be of advantage if simple models are used for external irradiation, from which the systematic effects can be modulated in order to compare these to the application of exposed radionuclides [38]. The concept of photodynamic therapy via Cherenkov light is promising. However, it must be better examined, explained, and understood before it could possibly be introduced into daily clinical routine.

## Data Availability

The data presented in this study are available on request from the corresponding author.

## Abbreviations

Re-188	Rhenium-188
SSB	Single strand break
PDT	Photodynamic therapy
CL	Cherenkov light
Ga-68	Gallium-68
Y-90	Yttrium-90
DMSO	Dimethyl sulfoxide
LET	Linear energy transfer
SC	Supercoiled (conformation)
OC	Open circular (conformation)
L	Linear (conformation)
SEM	Standard error of the mean
SD_pooled_	Pooled standard deviation
SF	Survival fraction
PUVA therapy	UVA therapy with psoralen
DSB	Double strand break
ALA	Aminoevulinic acid
F-18-FDG	Fluor-18-fluorodeoxyglucose
